# Synaptic dysfunction in ALS and FTD: anatomical and molecular changes provide insights into mechanisms of disease

**DOI:** 10.3389/fnmol.2022.1000183

**Published:** 2022-10-03

**Authors:** Pauline A. Gelon, Paul A. Dutchak, Chantelle F. Sephton

**Affiliations:** Department of Psychiatry and Neuroscience, CERVO Brain Research Centre, Laval University, Quebec City, QC, Canada

**Keywords:** amyotrophic lateral sclerosis (ALS; Lou Gehrig Disease), frontotemporal dementia (FTD), motor neuron disease (MND), synapse, dendrite, C9ORF72, TDP-43 (TAR DNA binding protein 43), fused in sarcoma (FUS)

## Abstract

Synaptic loss is a pathological feature of all neurodegenerative diseases including amyotrophic lateral sclerosis (ALS) and frontotemporal dementia (FTD). ALS is a disease of the cortical and spinal motor neurons resulting in fatal paralysis due to denervation of muscles. FTD is a form of dementia that primarily affects brain regions controlling cognition, language and behavior. Once classified as two distinct diseases, ALS and FTD are now considered as part of a common disease spectrum based on overlapping clinical, pathological and genetic evidence. At the cellular level, aggregation of common proteins and overlapping gene susceptibilities are shared in both ALS and FTD. Despite the convergence of these two fields of research, the underlying disease mechanisms remain elusive. However, recent discovers from ALS and FTD patient studies and models of ALS/FTD strongly suggests that synaptic dysfunction is an early event in the disease process and a unifying hallmark of these diseases. This review provides a summary of the reported anatomical and cellular changes that occur in cortical and spinal motor neurons in ALS and FTD tissues and models of disease. We also highlight studies that identify changes in the proteome and transcriptome of ALS and FTD models and provide a conceptual overview of the processes that contribute to synaptic dysfunction in these diseases. Due to space limitations and the vast number of publications in the ALS and FTD fields, many articles have not been discussed in this review. As such, this review focuses on the three most common shared mutations in ALS and FTD, the hexanucleuotide repeat expansion within intron 1 of *chromosome* 9 *open reading frame 72* (*C9ORF72*), *transactive response DNA binding protein 43* (*TARDBP or TDP-43*) and *fused in sarcoma* (*FUS)*, with the intention of highlighting common pathways that promote synaptic dysfunction in the ALS-FTD disease spectrum.

## Introduction

Amyotrophic lateral sclerosis (ALS) is a fatal disease that stems from the degeneration of both upper cortical and lower spinal motor neurons resulting in dysfunction of voluntary muscles. In 1874, French neurologist Jean-Martin Charcot named the disease based on post-mortem observations that muscles lacked nourishment or were “atrophic” and the “lateral” regions of the spinal cord were hardened or had “sclerosis”. Our understanding of ALS has evolved from the simplistic view that the degeneration of motor neurons cause the disease, to the recognition that ALS is a multisystem neurodegenerative disease involving other neuron populations and regions in the brain and spinal cord, glial and immune cells as well as peripheral tissues, which contribute to the disease (Tortarolo et al., 2017; Grossman, 2019). ALS is a heterogenous disease regarding age of onset, site of onset, disease progression, as well as pathological characteristics. ALS patients are clinically classified as having classical or atypical ALS, based on the level of motor neuron involvement and pattern of onset (Grad et al., 2017). From the time an individual first experiences symptoms of ALS, their mean survival time is 3–5 years (Brown and Al-Chalabi, 2017). The global number of ALS cases per year is approximately 2.1–4.4 per 100,000 population (Chio et al., 2013; Xu et al., 2020) and the cumulative lifetime risk in the United States and Europe is one in 400 (Brown and Al-Chalabi, 2017). Sporadic ALS represents 90% of cases, with ~10% of ALS cases having a family history of the disease. In most cases, the mean age of onset for those who develop ALS is between the 55 and 75 years, with an average age of diagnosis at 55 years (Ingre et al., 2015). However, juvenile (<25 years old) and “young-onset” ALS cases (<45 years old) represent ~1% and 10% of all cases, respectively (Turner et al., 2012). In sporadic ALS the ratio of affected males to affected females is 2:1, and in familial ALS the ratio is approximately 1:1 (Brown and Al-Chalabi, 2017). Currently, there are no therapies or effective treatments for ALS, thus the need for a deeper understanding of this disease is greatly needed to develop targeted therapeutic strategies.

Frontotemporal dementia (FTD) was first described in 1892 by Arnold Pick as a form of dementia characterized by a progressive neuronal loss, primarily across the frontal and temporal lobes, which leads to changes in executive functions, personality, abnormal behaviors and language impairments (Kurz et al., 2014; Mulkey, 2019). Like ALS, FTD is also recognized as a heterogeneous clinical, pathological and genetic disorder and is broadly referred to as frontotemporal lobar degeneration (FTLD) due to the region-specific neuronal atrophy that occurs in the brain. Based on a patient’s clinical presentation, FTD is divided into three main subtypes that include behavioral variant (bvFTD), semantic dementia (svPPA), and progressive non-fluent aphasia (nfvPPA) (Olney et al., 2017; Devenney et al., 2019). From the time symptoms of FTD first appear, the mean survival time is 3 and 17 years (Olney et al., 2017; Devenney et al., 2019). The annual incidence of FTD is approximately 1.6–4.1 cases per 100,000 population per year (Onyike and Diehl-Schmid, 2013; Coyle-Gilchrist et al., 2016). Over half of FTD cases are sporadic, but ~40% have family histories of dementia, cognitive or motor disorder (Olney et al., 2017; Devenney et al., 2019). FTD is the most common type of dementia in individuals under the age of 65 with a mean age of onset at 52.8 years with the affected ratio of males to affected females being 14:3 (Ratnavalli et al., 2002). Similar to ALS, there are no effective treatments for FTD.

ALS and FTD are part of a common disease spectrum and share clinical, pathological and genetic features. In addition to well-characterized motor defects of ALS, ~50% of ALS patients have behavioral changes and cognitive abnormalities that are linked with degeneration of the frontal and temporal lobes, and are classified as ALS-FTD (Lomen-Hoerth et al., 2002). Among FTD subtypes, ~40% of patients with bvFTD develop motor neuron dysfunction and meet the criteria for motor neuron disease and are classified as FTD-MND (Lomen-Hoerth et al., 2002; Lipton et al., 2004). Moreover, ~50% of patients with MND develop varying forms of cognitive impairment (Lomen-Hoerth et al., 2003).

Landmark discoveries showing the RNA-binding protein, transactive response DNA binding protein 43 (TDP-43 or TARDBP), as the main component of ubiquitinated inclusions in both familial and sporadic forms of ALS and FTD helped establish new models of protein-aggregate dependent neuronal dysfunction (Arai et al., 2006; Neumann et al., 2006). Since this time, TDP-43 aggregation is now considered to be a general pathological marker for ALS and FTD (Saberi et al., 2015), as well as Alzheimer’s disease (Josephs et al., 2015), Parkinson’s disease (Nakashima-Yasuda et al., 2007), traumatic brain injuries (Johnson et al., 2011) and within the normal aging brain (Uchino et al., 2015). Nonetheless, the seminal discovery that TDP-43 mislocalization and aggregation occurs in both ALS and FTD brought forth novel research aimed at uncovering the overlapping mechanisms by which these diseases occur. Subsequently, fused in sarcoma (FUS) accumulation was identified in neurons and glia in familial ALS cases with gene mutations in FUS (Kwiatkowski et al., 2009; Vance et al., 2009). Following these studies, FUS aggregates were also identified in affected brain regions of patients with sporadic forms of FTD in the absence of FUS mutations (Neumann et al., 2009). In 2011, toxic accumulation of dipeptides repeat (DPR), which can be translated from a hexanucleotide repeat expansion of the GGGGCC (G_4_C_2_) nucleotides within intron 1 of *chromosome* 9 o*pen reading frame 72* (*C9ORF72*), were identified in familial ALS and FTD cases with these gene expansions (DeJesus-Hernandez et al., 2011; Renton et al., 2011).

To date, there are at least 17 common genes that have been linked to the susceptibility of familial forms of ALS and FTD (Table 1) (Al-Saif et al., 2011; Belzil et al., 2013; Liscic et al., 2020; Anoar et al., 2021). Hexanucleotide repeat expansions within *C9ORF72*, mutations in *TARDBP* (*or TDP-43*) and *FUS* represent the majority of autosomal dominant mutations in familial ALS (Liscic et al., 2020; Anoar et al., 2021). Whereas *C9ORF72*, *progranulin* (*GRN)*, and *microtubule-associated protein tau* (*MAPT)* represent the majority of autosomal dominant mutations found in familial FTD (Liscic et al., 2020; Anoar et al., 2021). Based on the most frequent overlapping genes and pathological protein deposits of ALS and FTLD, these diseases can be further classified as being ALS-TDP, ALS-FUS, ALS-C9 or FTLD-TDP, FTLD-FUS, FTLD-C9. While it is unlikely that a single gene is solely responsible for causing sporadic ALS and FTD, the data suggest that these pathologies are the result of alterations of multiple genes that are found in a common or related pathways, which collectively contribute to the neurodegenerative outcome (Liscic et al., 2020). Indeed, the common features of ALS and FTD reinforce this concept, but more work is needed in order to identify the primary mechanism(s) of disease.

**Table 1:** ALS and FTD disease-associated gene mutations, their functions, and reported effects on neuromorphology and synapses.

**Gene**	**Encoding protein**	**Known protein function**	**Neuronal morphology and synapses**				**Sources**
			**Dendrites**	**Spines**	**Synapses**	**NMJs**
*ATXN2*	ATXN2	RNA metabolism; DNA repair	✓	✓	✓	ND	Arsovic et al. (2020)
*C9ORF72*	C9ORF72	Protein trafficking, proteostasis; vacuolar transport; mitochondria, oxidative stress	✓	✓	✓	✓	Ho et al. (2019), Lall et al. (2021), and Huber et al. (2022)
*CCNF*	CCNF	Protein trafficking and proteostasis	ND	ND	ND	ND	Not reported
*CHCHD10*	CHCHD10	Mitochondria and oxidative stress	ND	ND	✓	✓	Woo et al. (2017), Anderson et al. (2019), and Xiao et al. (2020)
*CHMP2B*	CHMP2B	Protein trafficking, proteostasis; vacuolar transport	✓	✓	✓	✓	Belly et al. (2010), Chassefeyre et al. (2015), and Waegaert et al. (2022)
*DCTN1*	DCTN1	Axo-dendritic transport	✓	ND	✓	✓	Yu et al. (2018) and Bercier et al. (2019)
*FUS*	FUS	DNA/RNA binding protein, RNA metabolism, transcription; DNA repair	✓	✓	✓	✓	Fujii et al. (2005), Sephton et al. (2014), Qiu et al. (2014), Udagawa et al. (2015), Shiihashi et al. (2017), Tibshirani et al. (2017), Picchiarelli et al. (2019), and Ho et al. (2021)
*OPTN*	OPTN	Protein trafficking, proteostasis; vacuolar transport	ND	ND	ND	ND	Not reported
*SIGMAR1*	SIGMAR1	Protein trafficking, proteostasis; metabolism	ND	ND	ND	ND	Not reported
*SQSTM1*	SQSTM1	Protein trafficking and proteostasis	ND	ND	ND	ND	Not reported
*TARDBP*	TDP-43	DNA/RNA binding protein; RNA metabolism; transcription; DNA repair	✓	✓	✓	✓	Majumder et al. (2012), Arnold et al. (2013), Handley et al. (2017), Herzog et al. (2017), Chand et al. (2018), Jiang et al. (2019), Wu et al. (2019), Herzog et al. (2020), Dyer et al. (2021a), and Ni et al. (2021)
*TBK1*	TBK1	Protein trafficking and proteostasis	✓	✓	✓	✓	Duan et al. (2019) and Sieverding et al. (2021)
*TIA1*	TIA1	Stress granule assembly; axo-dendritic transport	ND	ND	✓	ND	LeBlang et al. (2020)
*TREM2*	TREM2	Immune response	ND	ND	ND	ND	Not reported
*TUBA4A*	TUBA4A	Cytoskeletal dynamics	ND	ND	ND	✓	Buscaglia et al. (2020)
*UBQLN2*	UBQLN2	Protein trafficking and proteostasis	ND	✓	✓	✓	Gorrie et al. (2014) and Chen et al. (2018)
*VCP*	VCP	Protein trafficking, proteostasis; vacuolar transport	✓	✓	✓	✓	Shih and Hsueh (2016, 2018), Hall et al. (2017), and Huang et al. (2020)

Abbreviations: ND, not determined; ✓, gene reported to affect neuronal morphology or synapses in rodent or *in vitro* models; ATXN2, Ataxin 2; C9ORF72, Chromosome 9 Open Reading Frame 72; CCNF, Cyclin F; CHCHD10, Coiled-Coil-Helix-Coiled-Coil-Helix Domain Containing 10; CHMP2B, Charged Multivesicular Body Protein 2B; DCTN1, Dynactin Subunit 1; FUS, Fused in Sarcoma; OPTN, Optineurin; SIGMAR1, Sigma Non-Opioid Intracellular Receptor 1, SQSTM1, Sequestosome 1; TDP-43, TAR DNA Binding Protein; TBK1, TANK Binding Kinase 1; TIA1, T-cell Intracellular Antigen 1; TREM2, Triggering Receptor Expressed on Myeloid Cells 2; TUBA4A, Tubulin Alpha 4A; UBQLN2, Ubiquilin 2; VCP, Valosin Containing Protein.

While environmental and genetic risk factors are shown to be implicated in causing ALS and FTD, there is no consensus as to how these diseases originate (Al-Chalabi and Hardiman, 2013; Killin et al., 2016; Liscic et al., 2020). The identification of ALS and FTD-linked gene mutations in particular, have led to a multitude of studies that suggest mitochondrial function, cytoskeletal dynamics, protein homeostasis and axonal trafficking are implicated in causing these diseases (Abramzon et al., 2020; Castellanos-Montiel et al., 2020). Our understanding of the functional consequences caused by these defects are still ongoing, however, recent discovers made using *in vivo* and *in vitro* models of ALS/FTD strongly suggests that early synaptic dysfunction is a unifying hallmark of in these diseases. While cognitive impairments in ALS and motor defects in FTD have been reported in several early studies, the formal recognition of the convergent features of these diseases has only occurred recently, making the consolidation of the literature on ALS, ALS-FTD, and FTD-MND an inherent challenge. Here we provide a summary of the reported anatomical changes that occur in both cortical and spinal neurons in ALS, ALS-FTD, and FTD-MND. Additionally, we provide a summary of *in vitro* and *in vivo* models of ALS/FTD that report similar anatomical changes in motor neurons and their synapses. We describe recent work that identifies changes in the proteome and transcriptome in ALS/FTD models and provide a conceptual overview of the processes contributing to synaptic dysfunction in ALS and FTD. We also highlight recent work on C9ORF72, TDP-43, and FUS and their involvement in disrupting synaptic homeostasis.

## Neuropathological Features of ALS and FTD

Neurons are highly complex cells with elaborate dendritic branches that contain numerous spines and an axon that can extend long distances to form synapses at its terminal bouton. Motor neurons form a complex network of circuits that enable voluntary and involuntary movements through innervation of muscles. Motor neurons are grouped into upper motor neurons (UMNs), also known as Betz cells, located in the motor cortex, and lower motor neurons (LMNs), located in the brainstem and spinal cord (Brown and Al-Chalabi, 2017). UMNs are responsible for integrating excitatory and inhibitory inputs from the cortex and translating them into signals that will initiate or inhibit voluntary movement. They form local synaptic connections within the cortex, as well as distal connections in the brainstem and spinal cord. The majority of UMN axons cross over the contralateral side in the brainstem and travel down the lateral corticospinal tract to form synapses directly with interneurons and lower motor neurons (LMNs), located in the anterior horn of the spinal cord. UMNs use the neurotransmitter, glutamate, to relay their signals and integrate excitatory and inhibitory inputs from the cortex to their synaptic targets. LMNs directly or indirectly innervate effector muscles at the neuromuscular junction (NMJ) *via* synaptic release of acetylcholine. Together, LMNs, muscle fibers and NMJs form a motor unit. LMNs include visceral motor neurons that contribute to both the sympathetic and parasympathetic functions of the autonomic nervous system and somatic motor neurons. Somatic motor neurons project their axons to skeletal muscles and are responsible for movement. They are further divided into alpha, beta and gamma motor neurons according to the muscle fiber they innervate within a specific muscle target (Zayia and Tadi, 2022).

The extent to which UMNs and LMNs are affected in ALS and FTD-MND can vary between individuals but the extent to which they are affected correlates with the type of motor dysfunction presented. When UMNs fail, there are signs of muscle stiffness and spasticity. When LMNs fail, muscle twitching or fasciculations occur as they degenerate and the muscles that they innervate atrophy (Brown and Al-Chalabi, 2017). In ALS, there is selective vulnerability of the alpha motor neurons. Among the first to degenerate are the fast fatiguing (FF) motor units, which are the largest motor units and innervate the fast-contracting and fatigable muscle fibers (FF type). This is followed by degeneration of the fast fatigue-resistant motor units, which innervate the fast-contracting and fatigue-resistant muscle fibers (FR type). Interestingly, the most resistant and least affected are the slow (S) motor units, which mainly innervate the slow contracting and fatigue-resistant fibers (S type) (Ragagnin et al., 2019). Collectively, it is the degeneration of the corticospinal tract and its motor units that leads to NMJ denervation, muscle atrophy, and loss of motor function. In classical ALS, which involves both UMNs and LMNs, facial muscles and extremities are mainly affected. Clinical subsets of ALS can be distinguished further by the anatomical location of disease onset. This includes bulbar onset where symptoms first appear in the muscles that control speech, mastication and swallowing due to onset of disease in motor neurons located in the brainstem. As well as limb or spinal onset, where symptoms initially present in the upper or lower limbs because of disease onset in UMNs and LMNs. Patients with bulbar onset have a much worse prognosis than those with spinal onset ALS, with an average survival time of <2 years following a diagnosis (Turner et al., 2010). Conversely, atypical forms of ALS, which involve either UMN or LMN, show spastic paraplegia, autoimmune disease, or demyelinating LMN disease, frequently with extended survival rates (Grad et al., 2017). Two other motor neuron diseases that should be mentioned are primary lateral sclerosis (PLS) with predominantly UMN involvement and comorbidities with FTD (Grace et al., 2011) and progressive muscular atrophy (PMA), which has predominant LMN involvement (Grad et al., 2017). Currently, these diseases are classified separately from ALS, however, it remains unclear if they are discrete disorders or variants of ALS and FTD-MND (Grad et al., 2017). In FTD-MND there is preferential degeneration of neurons in the frontal and temporal lobes, but there is also degeneration of UMNs and LMNs, which coincides with impaired motor function (Devenney et al., 2019). It is unclear why motor neurons are particularly vulnerable to degeneration in ALS and FTD-MND and it is equally unclear why some motor neurons are spared while other degenerate (Burrell et al., 2011; Nijssen et al., 2017).

The diagnosis of ALS, ALS-FTD, and FTD-MND is based on criteria that use clinical, electrophysiological and neuropathologic examination to assess motor and cognitive dysfunction. Diagnosing ALS is based on the El Escorial revised criteria (Brooks et al., 2000), to assess LMN and UMN degeneration and help rule out other diseases or processes involved in motor neuron dysfunction. Following the classification motor neuron disease, subsequent clinical criteria defines cognitive and behavioral dysfunctions that are used to diagnose ALS-FTD, based on criteria developed by Strong et al. (2009). These criteria require that an individual diagnosed with ALS meets at least two non-overlapping supportive diagnostic features from either the Neary and/or Hodge criteria for the diagnosis of FTD (Strong et al., 2009). The diagnosis of FTD using the Neary and Hodge criteria are defined by early decline in social and personal conduct, emotional blunting, loss of awareness and changes in verbal communication (Neary et al., 1998; Hodges and Miller, 2001). Following a diagnosis of FTD, clinical classification of FTD with motor neuron disease (FTD-MND) needs to meet the criteria of UMN and LMN dysfunction as defined by the El Escorial revised criteria (Geevasinga et al., 2016). However, due to the heterogeneity of symptoms experienced by individuals with ALS, ALS-FTD, and/or FTD-MND, it remains difficult to provide a definitive diagnosis of these diseases, especially in early disease stages when symptoms first appear.

There is growing evidence from ALS patients, FTD patients, and animal models that suggest synaptic dysfunction begins very early in the disease, before symptom onset and motor neuron death. Studies using electromyography (EMG), nerve conduction velocity (NCV), and transcranial magnetic stimulation (TMS) to examine the electrophysiological properties of LMNs show that ALS patients in the early stages of disease already have indications of corticospinal degeneration, loss of LMNs and altered excitability of surviving motor units (Dengler et al., 1990; Zanette et al., 2002; Vucic and Kiernan, 2006; Marchand-Pauvert et al., 2019). Comparable studies conducted in FTD-MND patients show similar abnormalities in central motor circuits (Alberici et al., 2008; Burrell et al., 2011; Coon et al., 2011) and can also be observed in FTD patients without clinical evidence of motor involvement (Alberici et al., 2008; Burrell et al., 2011; Coon et al., 2011). Investigation into the changes in corticospinal connectivity in ALS models indicate that excitability of motor neurons shifts from a hyperexcitable (Wainger et al., 2014) to a hypoexcitable state (Devlin et al., 2015; Naujock et al., 2016; Sommer et al., 2022) while NMJs are still functional (Martinez-Silva et al., 2018). The cause of changes in LMN excitability in ALS and FTD are under investigation, but may involve changes in synaptic inputs from UMNs (Pradhan and Bellingham, 2021). The idea that synaptic dysfunction occurs early in ALS, is further supported by post-mortem analysis of tissues from a 58-year-old ALS patient in the early stages of the disease who died unexpectedly (Fischer et al., 2004). This individual had muscle fibers that were grouped and angulated, which are consistent with acute and chronic denervation and reinnervation of the muscles by LMN synaptic terminals (Fischer et al., 2004), consistent with observations in pre-symptomatic ALS mouse models (Chand et al., 2018; Martineau et al., 2018). Importantly, there was little axon degeneration in the spinal cord, the corticospinal tract was intact with no degeneration of UMNs or LMNs and there was no activation of glial cells (Fischer et al., 2004). While degeneration of motor neurons is a defining pathological feature of ALS and FTD-MND, these findings strongly suggest that motor neuron loss and denervation of NMJs occurs late in the disease process.

**Figure 1:**
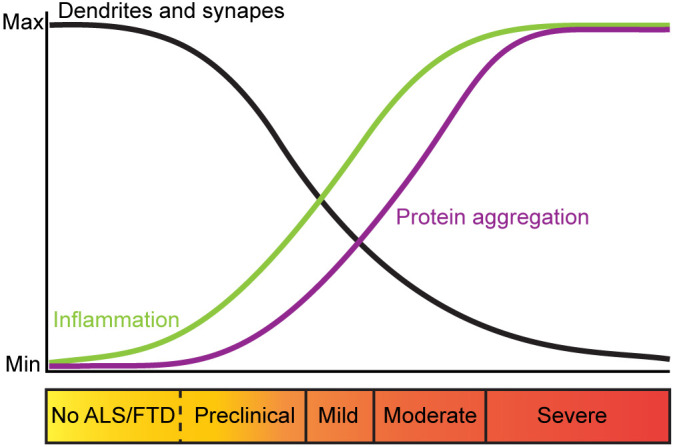
Trajectory of pathological changes and corresponding clinical presentation in ALS and FTD. Trajectory of changes in dendritic branches and synapses in ALS and FTD (black), inflammation (green) and protein aggregation (purple) relative to clinical symptoms of ALS and FTD. In ALS and FTD, dendritic branches and synapses are lost prior to clinical onset of disease, suggesting that perturbed neuronal morphology and synaptic maintenance are mechanisms that trigger synaptic loss and neurodegeneration associated with these diseases.

There is evidence that alterations in dendritic branching and dendritic spines are an early pathological feature of ALS and FTD-MND. Indeed, dendritic length, complexity and branch thickness are indicators of a neuron’s health and connectivity (Kulkarni and Firestein, 2012; Kweon et al., 2017). Post-mortem Golgi staining and histological analysis of intact motor neurons from ALS and FTD-MND brain and spinal cord tissues show dendritic attrition and thinning of dendritic branches in the apical and basal dendrites of UMNs (Hammer et al., 1979; Horoupian et al., 1984; Ferrer et al., 1991; Genc et al., 2017) as well as LMNs of patients with motor defects (Kato et al., 1987). Consistent with the alterations in dendrites in ALS and ALS-FTD, synaptic integrity is also affected. In sporadic ALS, the pre-synaptic densities of UMNs are reported to be unchanged, however, the number of post-synaptic densities are significantly reduced (Genc et al., 2017; Henstridge et al., 2018). These findings are consistent with reports that pre-synaptic densities around the soma and proximal dendrites of the LMNs are significantly decreased in ALS spinal cord tissues (Sasaki and Maruyama, 1994). These observations support the hypothesis that ALS starts in the UMNs, descends to the LMNs and progresses to extra-motor brain regions (Braak et al., 2013; Brettschneider et al., 2013). In FTD and FTD-MND, synaptic loss, reduction in the number of spines (Liu et al., 1996; Ferrer, 1999; Lipton et al., 2001), and synaptic densities in the cortex have also been reported (Brun et al., 1995; Liu et al., 1996; Ferrer, 1999). Recent imaging techniques have been developed to study synaptic density in living FTD patients and support these histological findings. The use of [11C]UCB-J PET, a radio-ligand that selectively binds to synaptic vesicle protein 2A (SV2A), to assess the regional distribution of synaptic loss in patients with probable bvFTD and carriers of C9ORF72 hexanucleotide repeat expansions, showed that loss of synaptic densities occur in the frontotemporal region (Malpetti et al., 2021; Salmon et al., 2021). Importantly, this study showed that pre-symptomatic FTD-C9 patients had reduced synaptic density and that extensive synaptic loss was observed in patients with severe cognitive impairments (Malpetti et al., 2021). These findings are consistent with sporadic ALS patients that showed synaptic loss correlated with severity of cognitive impairments and not due to cortical atrophy (Henstridge et al., 2018). While dendritic spines of LMNs are not readily examined in ALS and FTD-MND patient studies, changes in LMN spines are found to occur in the pre-symptomatic stages of disease an ALS mouse model (Fogarty et al., 2020, 2021). Thus, characterization of LMN spines in ALS and FTD-MND may also be relevant to our understanding of corticospinal connectivity and disease. Collectively, these observations further support the view that dendritic attrition and synaptopathy are early markers of ALS and FTD and that synaptic defects occur in the corticospinal tracts prior to the clinical presentation of motor dysfunction, inflammation and motor neuron death (Figure 1).

## Genetic Links to Synaptic Dysfunction

Synaptopathy can occur due to changes including altered Ca^2+^ levels at synapses, altered protein expression within the synapse, and dysfunctional neurotransmitter cycling (Wishart et al., 2006; Lepeta et al., 2016; Fogarty, 2019). Concomitant with synaptopathy in ALS and FTD are abnormalities in the cytoskeleton, mitochondrial dysfunction and accumulation of misfolded proteins (Wishart et al., 2006; Sasaki and Iwata, 2007; Lepeta et al., 2016; Fogarty, 2019; Gao et al., 2019; Castellanos-Montiel et al., 2020). However, it is unclear how changes in these cellular processes trigger the cascade of events that lead to synaptic loss in ALS and FTD or whether changes at the synapse promote wide-spread neuronal dysfunction.

Motor neurons have complex dendritic branches and long axonal projections, which rely on the integrity of cytoskeletal proteins for maintaining structural stability and transport of mitochondria, proteins and RNA within a neuron (Konietzny et al., 2017; Theunissen et al., 2021). As such, cytoskeletal changes within motor neurons make them vulnerable to synaptic dysfunction and neurodegeneration. Several gene mutations that encode proteins implicated in cytoskeletal dynamics and axonal transport have been identified in familial ALS or FTD, including: *dynactin subunit 1* (*DCTN1*) and* tubulin alpha 4a* (*TUBA4A*) (familial ALS and FTD) and *kinesin family member 5A* (*KIF5A*), *myelin associated oligodendrocyte basic protein* (*MOBP*) and *profilin 1* (*PFN1*) (familial ALS) and *MAPT* (familial FTD) (Brown and Al-Chalabi, 2017; Olney et al., 2017; Devenney et al., 2019; Liscic et al., 2020; Theunissen et al., 2021). Genetic changes of these proteins reviewed in detail elsewhere, affect cytoskeleton dynamics, impair dendrite and axonal integrity, which would affect transport of mitochondria, proteins and RNA to the synapse (Castellanos-Montiel et al., 2020).

Mitochondria are critical for maintaining synaptic integrity. Mitochondria are situated within dendrites, at the base of dendritic spines and in the post-synaptic compartments to provide energy in the form of ATP, synthesis of glutamate, and modulate Ca^2+^ levels to regulate synaptic transmission (Waagepetersen et al., 2003; Li et al., 2004; Devine and Kittler, 2018; Giandomenico et al., 2022). The mitochondria produce more than 90% of neuronal ATP (Harris et al., 2012), which is required in many steps of the synaptic vesicle cycle (Chua et al., 2010; Sudhof and Rizo, 2011) and protein synthesis (Pontes et al., 2015). Failure of proper mitochondria trafficking, function and positioning in dendrites and synapses have been observed in ALS and FTD (Okamoto et al., 1990; Sasaki and Iwata, 2007) and may contribute to early synaptic loss in disease (Gao et al., 2019). The importance of proper mitochondrial function in synaptic regulation is supported by mutations in genes linked to mitochondrial function including: *superoxide dismutase 1* (*SOD1*) for familial ALS and *coiled-coil-helix-coiled-coil-helix domain containing 10* (*CHCHD10*), *C9ORF72*, *FUS*, and *TARDBP* for both familial ALS and FTD (Chaussenot et al., 2014; Williams et al., 2016; Lau et al., 2018; Smith et al., 2019; Liscic et al., 2020).

**Figure 2:**
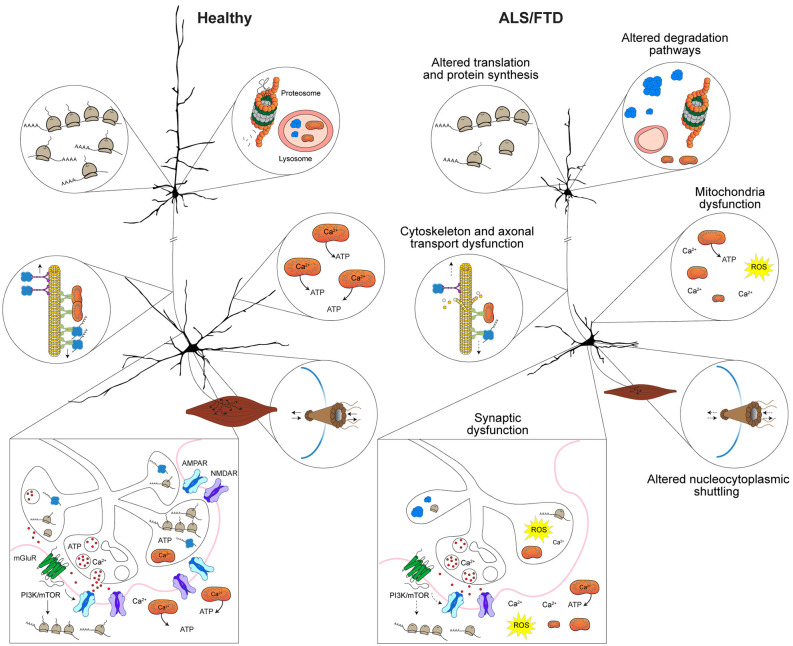
Mechanisms implicated in synaptic dysfunction in ALS and FTD. Altered proteostasis in ALS and FTD caused by misregulation of translation and protein synthesis affects several cellular processes including those implicated in neuronal morphology and synaptic homeostasis. Protein degradation pathways that involve the lysosome, autophagy, mitophagy, and the proteosome are dysfunctional in ALS and FTD result in toxic protein accumulation and lead to defects in neuronal function and synaptic communication. Cytoskeletal disruption or disarrangement that occur in ALS and FTD lead to structural instability of dendrites, axons and synapses and affect axonal transport of RNA granules, ribosomal subunits and organelles (i.e., mitochondria) to dendrites and synapse. In ALS and FTD, altered nucleocytoplasmic trafficking of proteins and altered physical properties of proteins (i.e., stress granule dynamics or liquid-liquid phase transitions) affect protein interactions and impact mRNA transport and local translation in axons, dendrites and synapses. Depletion or defective mitochondria within dendrites and at synapses that is observed in ALS and FTD affects Ca^2+^ levels and reduce ATP levels, which affects several processes including synaptic vesicle cycling and release, synaptic transmission, and local protein synthesis. Depicted upper and lower motor neurons were modified with permission from Sephton et al. (2014).

Due to the dynamic nature of the local synaptic environment, tight regulation of protein homeostasis can also impact several aspects of neuronal morphology and synaptic strength (Lottes and Cox, 2020; Giandomenico et al., 2022). As such, dendrites and synapses require some proteins to be transported from the cell body, however, they cannot rely on transport alone to maintain proteostasis (Lottes and Cox, 2020; Giandomenico et al., 2022). Instead, dendritic protein quality control systems, including free ribosomes and dendritic endoplasmic reticulum (ER) tubules, facilitate local translation (Lottes and Cox, 2020). Protein chaperones, endosomes, lysosomes, autophagosome and proteosomes that maintain proteostasis are also situation locally in dendrites (Lottes and Cox, 2020). Some proteins produced in the dendrites are then trafficked to activated synapses (Lottes and Cox, 2020). Within pre- and post-synaptic compartments several key elements such as free ribosomes and protein chaperones contribute to local proteostasis (Gorenberg and Chandra, 2017; Giandomenico et al., 2022). Altered proteostasis and accumulation of misfolded proteins caused by defects in protein synthesis and protein clearance pathways provides important insights into defects are underlying in ALS and FTD. Indeed, mutations in several genes linked with proteostasis and autophagy including *charged multivesicular body protein 2B* (*CHMP2B*), *sequestosome 1* (*SQSTM1/p62*), *ubiquilin-2* (*UBQLN2*), and *valosin containing protein* (*VCP*) are linked with familial ALS and FTD (Liscic et al., 2020). Integral to protein synthesis is trafficking and local translation of mRNA, which is another process that is altered in ALS and FTD and shown to essential in the maintenance of neuronal morphology and synapses. This is underscored by identified ALS and FTD mutations in genes that encode proteins involved in RNA metabolism including *FUS, TARDBP, ataxin 2* (*ATXN2*), *and T-cell intracellular antigen 1* (*TIA1*) (Ross et al., 2011; Takada, 2015; Brown and Al-Chalabi, 2017; Hofmann et al., 2019; Rubino et al., 2019). Based on these observations, there are strong indications that diverse mechanisms can contribute to synaptic dysfunction and biological distinctions of ALS and FTD (Figure 2).

## ALS/FTD Models of Disease

The identification of familial mutations linked with ALS and FTD have led to the development of *in vivo* and *in vitro* models of disease. These models have enabled more detailed biochemical investigation concerning how and when neuronal morphology and synaptic dysfunction occurs in relation to behavioral and pathological phenotypes. In this section, we discuss the known biological and pathological functions of C9ORF72, TDP-43, and FUS, which represent three major genes associated with ALS and FTD. Additionally, we discuss proteomic and transcriptomic findings that provide insight into mechanisms of synaptic dysfunction that occur in ALS and FTD.

### C9ORF72: biological and pathological overview

The C9ORF72 locus is located on the reverse strand of chromosome locus 9p21.2. The gene includes two non-coding exons (1a and 1b) and 10 coding exons (from 2 to 11) that give rise to three coding variants. Alternative splicing of these RNA variants results in the production of two different protein isoforms: a 222-amino acid (aa) isoform (C9-short of 24 kDa) encoded by variant 1 and a 481-aa isoform (C9-long of 54 kDa) that is encoded by variant 2 and 3, which differ in their inclusion of the non-coding exon 1b or 1a, respectively (DeJesus-Hernandez et al., 2011; Renton et al., 2011; Gijselinck et al., 2012). The C9ORF72 human gene is highly conserved in primates and across different species, suggesting that C9ORF72 proteins have fundamental biological functions (Smeyers et al., 2021). C9ORF72 is ubiquitously expressed in all brain regions and the spinal cord (DeJesus-Hernandez et al., 2011; Renton et al., 2011; Rizzu et al., 2016). In mice, the protein is localized in different cellular compartments during development and remains diffuse in the nucleus and soma in differentiated cells (Atkinson et al., 2015). The C9ORF72 protein can be found in biochemically purified synaptic fractions of brain homogenates (Atkinson et al., 2015) mainly in the pre-synaptic compartments (Frick et al., 2018), but also the post-synaptic compartment (Xiao et al., 2019). Analysis of C9ORF72 structure and its protein binding partners indicate that C9ORF72 acts as a guanine nucleotide exchange factor (GEF) for both Rab- and Rho-GTPases. It forms a complex with SMCR8 and WDR41 (Sellier et al., 2016; Sullivan et al., 2016) to act as a GDP/GTP exchange factor for RAB3, RAB8a, and RAB39b (Sellier et al., 2016; Sullivan et al., 2016; Frick et al., 2018), suggesting it has functions in membrane trafficking, synaptic transmission, cytoskeleton modulation, and autophagy. *C9ORF72* also regulates actin dynamics (Sivadasan et al., 2016) and stress granules formation (Maharjan et al., 2017).

To date, the hexanucleotide repeat G_4_C_2_ in intron 1 of *C9ORF72* is the most frequent autosomal dominant mutation causing ALS and FTD (DeJesus-Hernandez et al., 2011; Renton et al., 2011). Variation in length and expression levels of G_4_C_2_ repeats play a significant role in disease manifestation. For instance, the pathogenic length of >30 hexanucleotide repeats is found in ~10% of all ALS patients (Fratta et al., 2015). The characteristic pathological features in both ALS-C9 and FTLD-C9 cases is region-specific neuronal loss, gliosis, as well as accumulation of expanded transcripts in nuclear RNA foci and cytoplasmic TDP-43 aggregates (DeJesus-Hernandez et al., 2011; Renton et al., 2011; Liu et al., 2019). Distinct pathologic mechanisms have been hypothesized for G_4_C_2_ expansion, including: (1) a loss-of-function due to haploinsufficiency of C9ORF72 protein expression; (2) a gain-of-function of the repeat RNA aggregates or RNA foci; and (3) aggregation of dipeptide repeats (DPRs), which are transcribed from G_4_C_2_ RNA repeats by repeat-associated non-ATG-initiated (RAN) translation (Ash et al., 2013; Mori et al., 2013; Mizielinska and Isaacs, 2014). Several metabolic, proteostasis and neuronal processes are known to be impaired in *C9ORF72*-mediated pathogenesis, including autophagy, lysosomal function, ubiquitin-proteasome system (UPS), unfolded protein response (UPR) and axonal transport (Alhindi et al., 2021; Nishimura and Arias, 2021; Smeyers et al., 2021), all of which are shown to be critical for cellular health, but also for the organization, structure, strength and function of the synapse.

### C9ORF72: synaptic dysfunction in ALS/FTD models of disease

While some mouse models of *C9orf72* ALS/FTD do not show any major phenotypes associated with the human disease (Batra and Lee, 2017), others display axon degeneration, denervation of NMJs and loss of motor neurons that correspond with changes in cognitive and motor function (Liu et al., 2016; Batra and Lee, 2017; Herranz-Martin et al., 2017; Riemslagh et al., 2021). Notably, global knockout mouse models of C9ORF72 do not develop motor deficits (Koppers et al., 2015; Atanasio et al., 2016; Sudria-Lopez et al., 2016; Dong et al., 2021), unlike models expressing G_4_C_2_ repeats. One study that characterized four unique mouse models of *C9orf72* ALS/FTD, showed disease severity correlated with G_4_C_2_ repeat length and expression (Liu et al., 2016), consistent with findings in ALS/FTD-C9 patients. A single copy of C9-37 short repeats expressed in mice under control of a bacterial artificial chromosome (BAC) promoter caused no disease phenotype, whereas BAC-C9-36/29, which expressed short repeats with increased copies triggered mild disease progression (Liu et al., 2016). BAC-C9-500/32 and BAC-C9-500, with transgenes expressing copies containing a mix of ~500 and 32 repeats or ~500 repeats alone, showed a stronger decline in motor function, which corresponded with axonal degeneration, degeneration of UMN and LMN and denervation of NMJs (Liu et al., 2016). End-stage BAC-C9-500 and BAC-C9-500/32 mice that progressed more rapidly had extensive motor neuron loss in the lumbar spinal cord, along with antisense RNA foci and DPRs accumulation in ALS/FTD affected brain and spinal cord regions (Liu et al., 2016). These observations are consistent with another study that developed a mouse model overexpressing 102 G_4_C_2_ repeats mediated by adeno-associated virus (AAV) that found 102 G_4_C_2_ repeats generated DPR pathology, NMJ abnormalities, enhanced apoptosis of neuron populations, corresponding with deficits in gait and cognitive function (Herranz-Martin et al., 2017). Similarly, Thy1(GA)_149_-CFP transgenic mice with poly-GA inclusions within the brainstem and spinal cord had motor deficits in the absence of motor neuron loss (Schludi et al., 2017). Evidence points to DPR-mediated synaptic defects prior to cell death. Mobile GA aggregates found within neurites caused a reduction in SV2A (also known as SV2) and altered Ca^2+^ influx and synaptic vesicle release (Jensen et al., 2020). While the extent of the nucleotide expansion in C9ORF72 correlates with disease severity, further research into the mechanisms that contribute to C9ORF72 phenotypes will reveal novel therapeutic strategies.

The characterization of dendritic branches or spines have not been conducted in most C9orf72 ALS/FTD mouse models (Batra and Lee, 2017). However, in a microglia specific knockout mouse model of C9orf72, behavioral defects in these mice were attributed decreased dendritic branching in the motor cortex, which coincide with enhanced cortical synaptic pruning and decreased cortical synaptic proteins (Lall et al., 2021). In the same study, they found that C9orf72KO cortical neurons cultured in the absence of microglia did not have any changes in spine densities, however, in the presence of C9orf72KO microglia there was a reduction in synaptic densities and an increase in the number of pre-synaptic (vGlut1) and post-synaptic (PSD-95) markers within C9orf72KO microglia (Lall et al., 2021). These observations suggest that microglia have a role in dysfunctional synaptic pruning and synaptic loss observed in C9-ALS/FTD patients.

Findings from other *in vitro* models demonstrate a cell autonomous effect of C9orf72 on neuronal morphology and synapses. Primary hippocampal neurons from C9orf72KO mice have reduced dendritic arborization and decreased spine density (Ho et al., 2019). Furthermore, expression of the long isoform of human C9orf72, but not the short isoform, rescued the dendritic arborization phenotypes of C9orf72KO neurons through restoration of autophagy in these neurons (Ho et al., 2019). Overexpression of C9-RAN proteins in primary neurons resulted in RNA foci and DPR proteins as well as reduced dendritic branching (May et al., 2014; Zhang et al., 2014; Huber et al., 2022). The effects of poly-GA on these changes were attributed to impairments of the proteosome (May et al., 2014) and ER stress (Zhang et al., 2014). In addition to changes in dendritic branching, overexpression of 66 G_4_C_2_ mediated by AAV in primary hippocampal neurons resulted in fewer mature or mushroom-type spines, and increased numbers of stubby and thin spines (Huber et al., 2022). These morphological changes corresponded with hyperexcitation due to susceptibility of neurons to excitotoxicity (Huber et al., 2022), similar to reports from induced pluripotent stem cells (iPSCs)-derived motor neurons (Selvaraj et al., 2018; Shi et al., 2018). The hyperexcitation phenotype in neurons expressing C9ORF72 variants may be caused by the increased activity of N-methyl-D-aspartate (NMDA) or α-amino-3-hydroxy-5-methyl-4-isoxazole propionic acid (AMPA) receptors (Shi et al., 2018; Huber et al., 2022) and/or functional synaptic alterations due to impaired pre-synaptic vesicle dynamics (Perkins et al., 2021). In line with this, loss of C9orf72 in mouse brain lead to a post-synaptic upregulation of in AMPA receptor subunit, Gria1 (also known as GluR1 or GluA1) expression (Shi et al., 2018; Xiao et al., 2019) concomitant with a loss of Smrc8 expression and a decrease in Rab39b expression (Xiao et al., 2019). Changes in AMPA and NMDA receptor expression are also found in both patient-derived iPSCs and C9ORF72 patient post-mortem spinal cord tissues (Devlin et al., 2015; Selvaraj et al., 2018; Shi et al., 2018). These findings are consistent with C9ORF72 having both a loss and gain of function causing enhance neuronal sensitivity to excitotoxicity (Devlin et al., 2015; Selvaraj et al., 2018; Shi et al., 2018; Dong et al., 2021; Huber et al., 2022). Excitotoxicity of motor neurons due to over-stimulation of glutamate receptors was first proposed in the late 70s (Olney, 1978). Since then, experimental evidence has shown that major contributing factors to altered excitability and excitotoxicity of neurons in ALS/FTD are persistent activation of NMDA and AMPA receptors, due to reduced levels of astrocytic glutamate transporters and changes in AMPA and NMDA receptor composition and function, which lead to excess Na^+^ and Ca^2+^ influx and neuronal cell death (Van Den Bosch et al., 2006; King et al., 2016). Future studies will need to examine the mechanisms by which C9orf72 causes susceptibility of the neurons to glutamate-induced excitotoxicity, the susceptibility of different neuron populations and the interplay between different cell-types to better understand how this contributes to disease in ALS/FTLD-C9.

### Mechanisms of synaptic dysfunction caused by C9ORF72

Comparative proteomic analysis of frontal cortical tissues from postmortem tissues of ALS, FTD, ALS-FTD, and ALS-C9 patients show these disease groups share a down-regulation of proteins associated with neurons and synapses and up-regulation of proteins associated with astrocytes and microglia (Umoh et al., 2018). Consistent with reports that C9ORF72 plays an important role in immune regulation (Burberry et al., 2016; O’Rourke et al., 2016; Sudria-Lopez et al., 2016), proteins associated with astrocytes and microglia were slightly enriched in ALS-C9 when compared to sporadic ALS (Umoh et al., 2018). Proteomic analysis of *in vitro* ALS/FTD-C9 models show that proteins involved in protein synthesis and protein degradation are predominantly affected (Hartmann et al., 2018; Lualdi et al., 2021). ALS-C9 patient-derived primary skin fibroblasts have altered expression of proteins involved in proteostasis including ribosomal proteins (RPL7 and RPL38), translation initiation and elongation factors (EIF3B, EIF4A1, EEF1A1, EEF1A2), members of the HSP90 family, proteasome 26S subunits and aminoacyl-tRNA synthetases (Lualdi et al., 2021). These findings are consistent with proteomic studies from primary mouse cortical neurons overexpressing poly-(PR)_175_, one of the five possible peptides produced *via* RAN translation, which show significant reduction in cytosolic ribosomal proteins that corresponded with a reduction in synaptic and axonal proteins (Hartmann et al., 2018). This study showed that poly-(GR)_149_, localized to the nucleolus and reduced ribosome levels and translation similar to poly-PR, suggesting that impaired ribosome biogenesis may cause changes to proteostasis and drive toxicity (Hartmann et al., 2018). In support of these findings, interactome analysis of poly-GR/PR revealed ribosomes and RNA-binding protein interactors (Hartmann et al., 2018), suggesting that sequestration of these factors by poly-GR/PR could somehow impair ribosome biogenesis and protein synthesis. The relevance of poly-GR/PR interactions with these proteins is not yet clear, however, ribosome biogenesis is critical for the production of functionally matured ribosomes that are important for dendritic growth and development (Slomnicki et al., 2016; Chau et al., 2018). These findings may be relevant for our understanding how C9ORF72 hexanucleotide repeat expansions can impact neuronal features. Other potentially relevant molecular pathways to neuronal morphology and synaptic function in ALS-C9 patient-derived primary skin fibroblasts include ER-phagosome crosstalk, vesicle trafficking, the Slit/Robo signaling pathway, glucose metabolism, mitochondrial transport and bioenergetics (Lualdi et al., 2021). Indeed, the role of C9ORF72 in mitochondria function has been previously discussed (Smith et al., 2019) and mitochondria defects and elevated levels of reactive oxygen species have been observed in C9ORF72 models (Dafinca et al., 2016; Onesto et al., 2016). However, the mechanism by which C9ORF72 variants cause these changes to occur and their impact on neuromorphology and synapses needs to be investigated further.

Consistent with proteomic discoveries, transcriptome profiles from C9ORF72 models show dysregulation of genes that are involved in vesicle transport pathways, endosomal trafficking, lysosomal function, proteostasis, as well as synaptic transmission and neural differentiation (Sareen et al., 2013; Prudencio et al., 2015; Dickson et al., 2019; Liu et al., 2020; Perkins et al., 2021; Szebenyi et al., 2021; Sommer et al., 2022). However, it is unclear if these changes are directly caused by C9ORF72 variants or by the secondary effects of other factors like TDP-43 nuclear depletion and protein aggregation (DeJesus-Hernandez et al., 2011; Renton et al., 2011; Liu et al., 2019). To address the specific effects of C9ORF72, Liu et al. (2020) examined the transcriptome of cells without TDP-43 pathology from post-mortem human ALS/FTD-C9 brains. Their findings revealed that a hexanucleotide repeat expansion in *C9ORF72* caused mild expression and splicing changes (Liu et al., 2020). The genes that showed expression differences were involved in synaptic vesicles fusion or formation, vesicle transport, endosomal trafficking, chaperone associated protein aggregation and DNA repair (Liu et al., 2020). The mechanism by which C9ORF72 causes changes in transcripts associated with these functions is not clear. One study examined mRNA stability in ALS-C9 fibroblasts and iPSCs and found that destabilization of ribosomal (RPL28, RPL38, RPS18) and mitochondrial transcripts (COX6B, COX6C-X1, COX5B, NDUFA1, NDUFA13) occurs, consistent with ALS/FTLD-C9 and sporadic ALS brain and spinal cord tissues (Tank et al., 2018). Pathways involving inflammation, focal adhesion and the cytoskeleton were significantly enriched among RNAs that had increased synthesis (Tank et al., 2018). The mechanism by which C9ORF72 destabilizes mRNA is not yet known, but may involve defects in nucleocytoplasmic transport of RNA or expanded *C9ORF72* repeat sequestration of essential RBPs that regulate RNA stability (Ash et al., 2013; Mori et al., 2013; Mizielinska and Isaacs, 2014). Nevertheless, the impact of destabilizing ribosomal, mitochondria and other transcripts is likely to have a significant impact on a number of important cellular processes, including those involved in the maintenance of neuronal morphology and synaptic integrity.

### TDP-43: biological and pathological overview

In humans, *TARDBP* is located at chromosomal locus 1p36.22 and is comprised of six exons, five of which encode a 43 kDa protein. The gene coding TDP-43 is highly conserved throughout evolution and is found in all higher eukaryotic species including *Drosophila* *melanogaster*, *Xenopus laevis*, and *Caenorhabditis elegans* (Wang et al., 2004; Ayala et al., 2005). TDP-43 is an RNA-binding protein that contains an N-terminal region, a nuclear localization signal (NLS), two RNA recognition motifs: RRM1 and RRM2, a nuclear export signal (NES), C-terminal low complexity domain composed of glutamine/asparagine-rich (Q/N) and glycine-rich regions (Dreyfuss et al., 1993; Ayala et al., 2008; King et al., 2012). TDP-43 is ubiquitously expressed and predominantly localized in the nucleus (Ayala et al., 2008). The protein is also found in cytoplasmic RNA granules, axons, dendrites as well as pre- and post-synapses (Majumder et al., 2012; Gopal et al., 2017; Khalfallah et al., 2018; Briese et al., 2020; Altman et al., 2021; Wong et al., 2021). Knockout of the *TARDBP* gene in mice is embryonic lethal and embryonic stem cells that do not express TDP-43 are cell-lethal (Sephton et al., 2010; Wu et al., 2010).

TDP-43 has roles in gene transcription and RNA metabolism, including pre-mRNA splicing, mRNA stability and mRNA transport, in addition to microRNA (miRNA) and long non-coding RNA (lncRNA) processing (Buratti and Baralle, 2010; Bjork et al., 2022). Using RNA immunoprecipitation assays nearly 30% of the transcriptome or more than 6,000 mRNA targets, have been identified to associate with TDP-43 (Polymenidou et al., 2011; Sephton et al., 2011; Tollervey et al., 2011; Xiao et al., 2011). Further supporting the role of TDP-43 in regulating RNA metabolism, mass spectrometry studies of purified TDP-43 protein complexes show that it functionally associates with RNA splicing factors and proteins involved in RNA transport (Freibaum et al., 2010; Sephton et al., 2011). Analysis of TDP-43 and its interaction with proteins and DNA/RNA targets indicate that it is an important regulator of transcription and translation of genes involved in neuronal development and synaptic regulation (Sephton et al., 2012; Sephton and Yu, 2015; Alhindi et al., 2021; Bjork et al., 2022).

Cytoplasmic inclusion bodies that consist mainly of hyperphosphorylated and ubiquitinated TDP-43 are a common pathological feature observed in 95% of ALS and 50% of FTLD (Arai et al., 2006; Neumann et al., 2006; Chen-Plotkin et al., 2010). Following their discovery, missense mutations in the *TARDBP* gene were identified in both familial and sporadic cases of ALS (Kabashi et al., 2008; Rutherford et al., 2008; Sreedharan et al., 2008; Van Deerlin et al., 2008; Yokoseki et al., 2008). To date, over 50 *TARDBP* mutations from patients with sporadic and familial ALS have been identified (Lattante et al., 2013), accounting for ~5% of familial ALS and ~1% of sporadic cases (Chio et al., 2020). At least 9 *TARDBP* mutations have been identified in patients with FTD and FTD-MND (Borroni et al., 2009, 2010; Gitcho et al., 2009; Kovacs et al., 2009; Chio et al., 2010; Gelpi et al., 2014; Synofzik et al., 2014; Floris et al., 2015; Moreno et al., 2015; Caroppo et al., 2016). Among the *TARDBP* mutations identified, the A382T missense mutation is the most frequently identified in FTD patients (Chio et al., 2010; Synofzik et al., 2014; Floris et al., 2015; Caroppo et al., 2016).

In patients that have *TARDBP* mutations, TDP-43 aggregation is the most prominent histopathological feature (Chen-Plotkin et al., 2010). In ALS and FTD-MND, TDP-43 aggregates are found throughout affected regions of the cortical, brainstem and spinal cord, with the distribution of protein aggregates corresponding to the clinical features for each disorder (Geser et al., 2009). Many of the TDP-43 mutations are located within the glycine-rich C-terminal domain and promote aggregation of the protein (Johnson et al., 2009; Dewey et al., 2011). Proposed pathologic mechanisms of *TARDBP* mutations include altered physical properties related to stress granule formation, liquid-liquid phase transitions, and nucleocytoplasmic shuttling dynamics, which contribute to altered proteostasis, transcription, and translation that can impact a number of cellular functions (Loganathan et al., 2019; Bjork et al., 2022; Carey and Guo, 2022).

### TDP-43: synaptic dysfunction in ALS/FTD models of disease

Several ALS/FTD mouse models of TDP-43 report that expression of ALS-linked mutations, cause brain atrophy, muscle denervation, motor neuron loss, and denervation of NMJs along with progressive motor impairments (McGoldrick et al., 2013; Picher-Martel et al., 2016; White et al., 2018; Ebstein et al., 2019; Huang et al., 2020; Alhindi et al., 2021). Mice overexpressing TDP-43Q331K or wild-type human TDP-43 (TDP-43WT), in the brain and spinal cord under control of the murine prion promoter (Prp), showed dose and age-dependent motor defects caused by mutant TDP-43, which corresponded with progressive motor axon degeneration, motor neuron death and denervation of NMJs (Arnold et al., 2013). Importantly, this study showed that these changes occurred in the absence of TDP-43 insoluble aggregates and nuclear loss of TDP-43 (Arnold et al., 2013). Further characterization of the TDP-43Q331K mice showed denervation of NMJs and impaired neurotransmission preceded defects in motor function and motor neuron loss (Chand et al., 2018). These findings corresponded with altered innervation of NMJs as well as the distribution of synaptic vesicle-related proteins at NMJs (Chand et al., 2018), indicative of ongoing synaptic remodeling. The observed phenotypes at the NMJ were found to be the result of defects in fusion and release of synaptic vesicles within the motor nerve terminal (Chand et al., 2018), consistent with the role of TDP-43 in stabilizing mRNAs required for synaptic vesicle release (Keihani et al., 2019).

Other reported changes in different TDP-43 mouse models involve alterations in dendritic branching and spines (Fogarty et al., 2016; Handley et al., 2017; Wu et al., 2019; Dyer et al., 2021a). Conditional knockout of TDP-43 in the mouse forebrain (TDP-43cKO) using the Camk2α promoter, showed that loss of TDP-43 in neurons caused dendritic attrition in layer V neurons UMN and loss of spines (Wu et al., 2019). These changes corresponded with brain atrophy, decreased long-term potentiation (LTP) in the hippocampus, and cognitive defects in 12-month-old mice, which were followed by motor defects (Wu et al., 2019). A hippocampal-specific knockout of TDP-43 showed similar changes to dendritic branches and spines in CA1 neurons along with decreased LTP and spatial memory, which were attributed to altered sortilin splicing and impaired brain-derived growth factor (BDNF) secretion (Tann et al., 2019). Reintroduction of sortilin or BDNF partially restored these phenotypes in TDP-43 knockout models (Tann et al., 2019). Thy1-YFP:Prp-TDP43A315T mice also showed significant reduction in dendritic branching and spine density in the layer V UMN prior to cognitive and motor dysfunction (Handley et al., 2017; Jiang et al., 2019). Morphological analysis of the spines in these mice revealed a significant impairment in the development of mature spines in the motor cortex and lowered synaptic transmission (Handley et al., 2017). Consistent with these findings, mice expressing TDP-43ΔNLS, a form of TDP-43 that is retained in the cytoplasm, in excitatory neurons under control of the Camk2α promoter, showed TDP-43ΔNLS, and not TDP-43WT expression, caused a significant loss in spine density in layer V UMN, with few mature spines and mostly thin spines (Dyer et al., 2021a). These changes corresponded with a decreased expression in AMPA receptor subunits, Gria1, Gria2, and Gria3, and NMDA receptor subunit 2A and hyperexcitability in layer V excitatory neurons of the motor cortex (Dyer et al., 2021b). While TDP-43 is shown to bind the mRNA of AMPA receptor subunits Gria2–4 (Sephton et al., 2011; Narayanan et al., 2013; Jiang et al., 2019), the mechanism by which cytoplasmic TDP-43 disrupts mRNA translation of ionic glutamate receptor subunits or other mRNAs related to changes in dendrites and spines remain undefined. Other mouse models of TDP-43 also exhibit changes in the excitability of motor neurons (Fogarty et al., 2016; Zhang et al., 2016), and the collective body of work suggests there are both extrinsic and intrinsic factors that contribute to these changes.

Consistent with mouse models, *in vitro* studies show dysregulation of TDP-43 causes defects in dendritic growth, maturation of spines and synaptic transmission (Majumder et al., 2012; Han et al., 2013; Herzog et al., 2017, 2020; Tibshirani et al., 2017; Jiang et al., 2019; Ni et al., 2021). Depletion of TDP-43 from primary hippocampal and cortical neurons caused a significant decrease in dendritic branches (Han et al., 2013; Herzog et al., 2017, 2020). In contrast, another report showed that TDP-43 depletion from primary hippocampal neurons resulted in an increase in the number of matured spines without affecting neuronal morphology (Majumder et al., 2012). These changes corresponded with increased levels of the active form of Rac family small GTPase 1 (Rac1), a known positive regulator of spinogenesis (Wiens et al., 2005), clustering of AMPA receptors on the dendritic surface as well as increased individual current amplitude and average amplitude of mEPSCs (Majumder et al., 2012). Changes in Rac1, GTP-Rac1, and Gria1 at the surface of the hippocampal neurons caused by TDP-43 knockdown were rescued with a Rac1 inhibitor or co-expression with wild-type TDP-43 (Majumder et al., 2012). The effect of TDP-43 depletion in dissociated hippocampal neurons differs from work using organotypic rat hippocampal slice cultures that showed TDP-43 knockdown reduced the spine density and function of mature synapses (Ni et al., 2021). Importantly, the effect on synapses caused by TDP-43 depletion were rescued by wildtype TDP-43, but not by ALS/FTLD-associated mutants (Ni et al., 2021). The discrepancies between these studies require further investigation, but may involve differences in developmental time-points, cell-types and expression levels of TDP-43. Nonetheless, these studies collectively point to important roles for TDP-43 in the development and maintenance of neuromorphology and synapses.

Indeed, the expression of ALS/FTD-associated TDP-43 mutants in primary neuron cultures provides evidence that TDP-43 variants negatively impact neuronal morphology. TDP-43WT, M337V or A315T mutants caused a significant decrease in dendritic branches (Herzog et al., 2020). These phenotypes could be partially rescued by overexpression of a constitutively active forms of calcium/calmodulin-dependent protein kinase IV (CaMKIV) and cAMP-response element binding protein (CREB) (Herzog et al., 2020), which are known regulators of dendritic growth (Redmond et al., 2002). Further examination of the TDP-43A315T mutant, showed a reduction in dendritic spines of pyramidal cortical neurons corresponded with decreased localization of AMPA receptor subunit Gria1 to synapses and decreased spine densities (Jiang et al., 2019). The functional consequences of these changes were reduced generation of action potentials in TDP-43A315T-expressing pyramidal neurons (Jiang et al., 2019). While AMPA and NMDA receptors are implicated in dendritic architecture and spines of neurons (Inglis et al., 2002; Espinosa et al., 2009), the mechanism by which TDP-43 alters the expression of AMPA and NMDA receptor subunits is still not known. Additionally, it is still not known how misregulation of other TDP-43 mRNA targets implicated in mitochondria transport, morphology and function, cytoskeleton dynamics, axonal transport, proteostasis and autophagy (Oberstadt et al., 2018; Gao et al., 2019; Wood et al., 2021) would impact neuronal integrity and synaptic function.

Dendritic attrition due to loss of intermediate and terminal branches are also observed in primary motor neurons expressing hTDP-43G348C, but not in primary motor neurons expressing hTDP-43WT (Tibshirani et al., 2017). These changes corresponded with a reduction in subunits of the chromatin remodeling complex, neuronal Brahma-related gene 1 (Brg1)-associated factor complex (nBAF) expression (Tibshirani et al., 2017). Dendritic defects were attenuated by co-expression of the critical nBAF subunit, Brg1 (Tibshirani et al., 2017). Importantly, this complex is shown to be important for neuronal differentiation, dendritic extension and synaptic function (Wu et al., 2007) and nBAF subunits are depleted in spinal motor neurons of sporadic ALS and ALS-C9 cases (Tibshirani et al., 2017). The different effects of TDP-43 variants vs. wild-type TDP-43 expression on neuromorphology is a potentially important observation and may provide clues to vulnerabilities of specific neuron populations and the molecular pathways involved.

### Mechanisms of synaptic dysfunction caused by TDP-43

TDP-43 regulates diverse cellular processes including those involved in cytoskeletal dynamics, mitochondrial function, energy metabolism, axonal transport, signaling pathways and proteostasis (Iridoy et al., 2018; Oberstadt et al., 2018; Umoh et al., 2018; Wood et al., 2021; Bjork et al., 2022). Unbiased proteomic analysis of postmortem frontal cortical tissues from individuals with ALS and FTD revealed a correlation between TDP-43 pathology and cognitive dysfunction and significant changes in proteins associated with RNA-binding (HNRNPA1, MATR3, and PFN1 and TDP-43 itself), synaptic transmission (MAP1B, MAP1A, DCTN1, discs Large MAGUK Scaffold Protein 4: DLG4/PSD-95, Tubulins: TUBB4B, TUBA1A), mitochondria (NADH:ubiquinone oxidoreductase core subunits: NDUFV1, NDUFS1, NDUFA2, NDUFA8, ATP Synthase F1 Subunit Alpha: ATP5A1), astrocytes (Glial fibrillary acidic protein: GFAP), and microglia (Moesin: MSN) (Umoh et al., 2018). Similar changes were also found in post-mortem spinal cords from individuals with ALS-TDP and ubiquitin-positive FTLD (FTLD-U) (Iridoy et al., 2018). The mechanistic causes for changes protein expression caused by TDP-43 dysregulation and their relationship to synaptic dysfunction and disease require further investigation.

Studies that have examined the pre-symptomatic effects of TDP-43 in animal models provide insight into potential mechanisms by which TDP-43 initiate synaptic dysfunction. Transcriptomic analysis of pre-symptomatic and symptomatic TDP-43 mouse models demonstrate that significant changes in pre-mRNA splicing and RNA expression occur mostly in symptomatic mice (White et al., 2018; Gordon et al., 2019; Wu et al., 2019; Huang et al., 2020). Transcriptome analysis of forebrain TDP-43cKO mice showed fewer changes in gene expression from pre-symptomatic (3-month-old) compared to symptomatic (12-month-old) mice, the latter had a greater number of down-regulated genes (Wu et al., 2019). Glial-specific markers, *Gfap*, *serpin peptidase inhibitor clade A member 3* (*Serpina3a*), and *complement components* (*C4a/C4b*), were constitutively up-regulated in both pre-symptomatic and symptomatic mice, whereas down-regulated genes in pre-symptomatic TDP-43cKO mice encoded proteins involved in synaptic, endosome, and autophagosome functions (Wu et al., 2019). The authors demonstrated that down-regulation of *Dlg3/Psd-95* and the encoding synapse associated protein (SAP102) protein, were reduced in the cortex and synaptosomes of TDP-43cKO mice in an age-dependent manner. Consistent with SAP10 role in regulating synaptic plasticity through NMDA receptor recycling (Chen et al., 2012), down-stream mediators of the NMDA receptor, CaMKIV, NMDA receptor subunit NR2b (or Grin2B), and phospho-Erk1/2, are also found to be altered in TDP-43cKO mice (Wu et al., 2019). Several of the signaling proteins identified by Chen et al. (2012), are shown to activate CREB (Shaywitz and Greenberg, 1999), a transcription factor well known for regulating dendritic branching (Redmond et al., 2002). The implications of TDP-43 on the CREB signaling cascade are further supported with the identification of CREB2 and upstream activators of CREB as punitive TDP-43 RNA targets (Herzog et al., 2020). Collectively, these studies highlight the possible role of TDP-43 in regulating mRNAs involved in the NMDA receptor/CREB signaling cascade, but the direct and indirect role of TDP-43 in regulating this pathway and the implications for neuronal morphology and synaptic function require more detailed investigation.

*In vitro* evidence shows that the effects of TDP-43 on dendrites and synapses are mediated through its interactions with RNA. TDP-43 truncation mutants or point mutations in the RRM1 and RRM2 regions, which lack RNA binding, do not cause defects in dendritic growth (Herzog et al., 2017) and are not able to rescue synaptic defects caused by TDP-43 depletion (Ni et al., 2021). Moreover, the role of TDP-43 in RNA trafficking and translation regulation are shown to be important in maintaining neuronal morphology and synaptic integrity (Majumder et al., 2016; Tank et al., 2018; Briese et al., 2020; Altman et al., 2021). For example, TDP-43 is involved in *Rac1* transport and translation in dendrites (Majumder et al., 2016), whereas depletion of TDP-43 alters *Rac1* mRNA transport within neurons, which in turn affects Rac1 expression (Chu et al., 2019). Activation of the Rac1 pathway stabilizes dendritic spines and recruits AMPA receptors to the postsynaptic membrane, enhancing excitatory synaptic transmission (Wiens et al., 2005; Majumder et al., 2012). The previously mentioned changes in AMPA expression at dendritic spines in TDP-43 disease models are consistent with a potential reduction in Rac1-mediated AMPA recruitment to spines. There is also evidence that shows TDP-43 affects protein expression at the level of translation prior to global misregulation of gene and protein networks (Briese et al., 2020; Altman et al., 2021). Axonal accumulation of TDP-43ΔNLS was found to cause NMJ disruption through inhibition of local protein synthesis (Altman et al., 2021). Specifically, TDP-43ΔNLS accumulation promoted Ras-GAP SH3-domain-binding protein 1 (G3BP1)-positive ribonucleoprotein (RNP) assembly and mRNA sequestration within RNPs, which lead to a reduction in nuclear-encoded mitochondrial proteins and respiratory chain complex proteins by suppressing protein translation (Altman et al., 2021). The three reported nuclear-encoded mitochondrial genes, *ATP5A1*, *Cox4i1*, and *Ndufa4*, found to be affected by TDP-43, had increased mRNA abundance but decreased protein expression (Altman et al., 2021). These findings suggest that TDP-43ΔNLS sequestration of mRNA leads to deleterious effects on protein synthesis. Additionally, depletion of TDP-43 in primary mouse motor neurons can lead to translation repression and defects in in mRNA transport (Briese et al., 2020). Loss of TDP-43 led to defects in the axonal localization of transcripts encoding components of the cytoskeleton, the translational machinery and transcripts involved in mitochondrial energy metabolism (Briese et al., 2020). In the somatodendritic compartment of TDP-43 knockdown motor neurons, there was an upregulation of transcripts associated with neuron projection and dendrite development, synapses and cytoskeleton (Briese et al., 2020). Unique to axons, was the down-regulation of transcripts encoding translation initiation factors (eukaryotic initiation factor 4A-II: Eif4a2) and ribosomal proteins (40 S ribosomal proteins S8 and S3: Rps8 and Rps3, respectively) (Briese et al., 2020). These changes corresponded with a reduced capacity for protein production in axons of TDP-43 depleted motor neurons (Briese et al., 2020). Transcripts down-regulated in both the somatodendritic and axon compartments were associated with mitochondrial function and energy production, which corresponded with a significant decrease in the number of functional mitochondria in axons of TDP-43 depleted motor neurons (Briese et al., 2020). These findings demonstrate pathological TDP-43 interferes with multiple mitochondrial pathways, including bioenergetics, as well as biogenesis, quality control, transport and fission and fusion dynamics (Gao et al., 2019). Alterations in transcript and protein levels in TDP-43 models of ALS/FTD may involve several aspects of RNA metabolism including RNA trafficking, stability or translation regulation or possibly by its direct association with translation machinery (Russo et al., 2017; Chu et al., 2019; Charif et al., 2020; Sidibe et al., 2021). Therefore, further investigation is required to understand the mechanisms by TDP-43 causes changes in mRNA and protein levels and the temporal impact of these changes on neuronal morphology and synaptic function.

### FUS: biological and pathological overview

Human *FUS* is located at the chromosomal locus 16p11.2 and is comprised of 15 exons, which encode a 53 kDa protein that migrates on SDS-PAGE between ~68–75 kDa. The gene encoding FUS is found in all higher eukaryotic species with homologs expressed in *Drosophila* *melanogaster*, *Xenopus laevis*, and *Caenorhabditis elegans*. Historically, FUS has also been called translated in liposarcoma (TLS) and is a member of the FET family of RNA-binding proteins that include Ewing sarcoma breakpoint region 1 (EWS) and TATA-box binding protein associated factor 15 (TAF15) (Tan and Manley, 2009). FUS contains a disordered N-terminal serine-tyrosine-glycine-glutamine (SYGQ)-rich region, followed by an arginine-glycine-glycine (RGG1) box in the middle of the protein, a central conserved RRM, and a zinc finger (ZnF) domain, that is flanked by RGG2 and RGG3 boxes and a proline-tyrosine nuclear localization sequence (PY-NLS) at the extreme C-terminal end (Lerga et al., 2001; Iko et al., 2004; Bentmann et al., 2012). FUS is ubiquitously expressed, predominantly localized in the nucleus of cells, and undergoes nucleocytoplasmic shuttling (Zinszner et al., 1997; Tsai et al., 2022). In the cytoplasm, FUS is observed within RNA granules and diffusely in non-RNA granules within the axons, dendrites, and synapses, prominently pre-synaptic within UMN and LMN (So et al., 2018; Deshpande et al., 2019; Sahadevan et al., 2021).

The nuclear functions of FUS include the regulation of transcription, splicing and DNA damage repair (Hoell et al., 2011; Ishigaki et al., 2012; Lagier-Tourenne et al., 2012; Rogelj et al., 2012; Schwartz et al., 2012; Mastrocola et al., 2013), whereas the cytoplasm functions include numerous roles in RNA transport and stability, miRNA processing, and translation regulation (Fujii and Takumi, 2005; Morlando et al., 2012; Udagawa et al., 2015; Kapeli et al., 2016; Kamelgarn et al., 2018; Sevigny et al., 2020). FUS can bind several thousand RNAs at coding, non-coding and 5’- and 3’-UTR regions (Hoell et al., 2011; Ishigaki et al., 2012; Lagier-Tourenne et al., 2012; Rogelj et al., 2012), mediated through its RRM, ZnF domain and three RGG boxes (Burd and Dreyfuss, 1994; Lerga et al., 2001; Iko et al., 2004; Ozdilek et al., 2017). In mouse brain, ~6,300 genes contain both TDP-43 and FUS binding sites, with overlapping binding sites in ~2,700 genes (Lagier-Tourenne et al., 2012). Functional analysis of FUS, its protein partners and DNA/RNA targets indicate that it is an important regulator of transcription and translation of genes that encode proteins that contribute to diverse cellular processes (Ishigaki et al., 2012; Lagier-Tourenne et al., 2012; Rogelj et al., 2012; Mastrocola et al., 2013; Sephton and Yu, 2015).

Over 50 mutations have been identified in the *FUS* gene that account for approximately 5–10% of familial ALS and ~0.4% of sporadic ALS (Kwiatkowski et al., 2009; Vance et al., 2009). Only a few *FUS* mutations have been identified in familial FTD (Van Langenhove et al., 2010; Huey et al., 2012). ALS and FTD-linked *FUS* mutations result in pathological aggregation of FUS, with varying levels of ubiquitination, in the cytoplasm and nucleus of neurons and glia throughout the affected regions of the cortex, brainstem and spinal cord (Kwiatkowski et al., 2009; Munoz et al., 2009; Vance et al., 2009; Suzuki et al., 2012). In contrast, most FTLD cases with FUS pathology (FTLD-FUS) show no evidence of mutations in the *FUS* gene (Seelaar et al., 2010; Nicolas et al., 2022). These cases usually present as bvFTD or FTD-MND and tend to effect younger individuals having more rapid disease progress (Perry et al., 2017). The majority of *FUS* mutations that have been identified are located within the C-terminal PY-NLS and are shown to affect the subcellular distribution of the protein (Bentmann et al., 2013). Mutations in the FUS 3’UTR have also been identified in familial ALS that increase FUS expression (Sabatelli et al., 2013). The proposed pathologic mechanisms of FUS misregulation involve mislocalization of the protein to the cytoplasm due to mutations in the PY-NLS. These defects alter physical properties of the protein involving aberrant stress granule dynamics and liquid-liquid phase transitions, which contribute to altered proteostasis, transcription and translation that impair dendrites and spine development (Sephton and Yu, 2015; Shang and Huang, 2016; Alhindi et al., 2021; Carey and Guo, 2022).

### FUS: synaptic dysfunction in ALS/FTD models of disease

Several mouse models of ALS/FTD-FUS display similar aspects of the human disease, including changes to neuronal morphology and synapses, as well as cognitive and motor defects (McGoldrick et al., 2013; Picher-Martel et al., 2016; Alhindi et al., 2021). Global transgenic mice expressing low levels human wild-type FUS (FUSWT) or FUSR521G under Cre-inducible control of the CAG promoter, developed deficits in motor function, NMJ denervation and inflammation without the loss of motor neurons, degeneration of motor axons or FUS aggregation (Sephton et al., 2014). Importantly, only FUSR521G mice had dendritic attrition of the layer VI-V UMN and LMN and reduced mature spines of layer VI-V UMN, which corresponded with cognitive and motor impairments (Sephton et al., 2014). Characterization of early synaptic changes in pre-symptomatic FUSWT mice showed mitochondrial abnormalities in the pre-synaptic motor nerve terminals lead to smaller NMJs, loss of synaptic vesicles and synaptophysin (Syp) expression in the absence of motor neuron loss (So et al., 2018). Changes in mitochondrial abundance have also been observed in ALS/FTD-FUS patients and other disease models (Tradewell et al., 2012; Deng et al., 2015; Tsai et al., 2020), which may occur due to aberrant interactions with mRNA or protein interactions that lead to global changes in protein synthesis (Nakaya and Maragkakis, 2018; Tsai et al., 2020; Salam et al., 2021). Future studies will need to focus on understand the mechanisms by which FUSWT and FUS variants affect mitochondrial function in ALS/FTD-FUS disease models.

Other studies that examine the cell-autonomous effect of FUS variants show that restricted expression of FUS in the brain and spinal cord of mice is sufficient to promote dendritic attrition of UMN and LMN, alterations of dendritic spines and NMJs as well as behavior impairments (Qiu et al., 2014; Ho et al., 2021). Consistent with a loss of corticospinal connectivity observed in ALS and FTD-MND patients (Whitwell et al., 2011; Renga, 2022), Prp-FUSR521C mice had loss of dendritic branching and mature spines in UMN and a corresponding reduction in the number and size of presynaptic terminals in spinal motor neurons (Qiu et al., 2014). Brain atrophy and age-dependent cognitive defects in Prp-FUSR514G transgenic mice were also found to cause decreased dendritic spine density and LTP in the hippocampus of these mice (Ho et al., 2021). Consistent with synaptic defects caused by mislocalization of FUS variants to the cytoplasm, mouse models expressing FUSΔNLS, a FUS mutant that is retained in the cytoplasm, have reduced dendritic branching and spine densities, that correspond with behavioral impairments (Shiihashi et al., 2017; Picchiarelli et al., 2019). (KI) mice heterozygous for *FusΔNLS/+* mice had endplate denervation, reduced surface of area and reduced neuromuscular transmission of NMJs by 1 month of age (Picchiarelli et al., 2019); defects that preceded motor phenotypes in these mice by 6 months of age (Scekic-Zahirovic et al., 2016). Mice expressing FUSΔNLS under the Thy1 promoter are shown to have FTD-associated cognitive defects including hyperactivity, social interactional deficits, and impaired fear memory retrieval, with decreased dendritic spine and synaptic density in the frontal cortex (Shiihashi et al., 2017). Importantly, the changes in neuronal morphology and synapses of are shown to precede neuronal loss (Shiihashi et al., 2017). Changes to synapses corresponded with significant decreases in mRNA levels for *AMPA* receptor subunits FUS knockin *Gria3* and *Gria4* (also known as *GluR*3 o*r GluA3* and *GluR*4 o*r GluA4*, respectively) (Shiihashi et al., 2017). The functional consequence of reduced synaptic densities in Thy1-FUSΔNLS mice was a significant reduction in the frequency, but not the amplitude, of mEPSCc in layer V pyramidal neurons (Shiihashi et al., 2017). These results indicate that the function of each excitatory synaptic input is intact, but the total number of excitatory synaptic inputs is reduced in these mice. The mechanism by which FUS causes changes to the AMPA receptor subunits were not assessed in this study. However, mRNA transport was found to be impaired by cytoplasmic FUS aggregates found within the frontal cortex and hippocampus of Thy1-FUSΔNLS mice (Shiihashi et al., 2017), consistent with other studies that show cytoplasmic FUS aggregates alter translation through aberrant sequestration of RNA (Kamelgarn et al., 2018; Lopez-Erauskin et al., 2018). FUS depletion specifically in the hippocampus of mice causes delayed dendritic spine maturation and cognitive defects including hyperactivity, disinhibition and changes in social interactions (Udagawa et al., 2015). While global knockout of FUS leads to postnatal lethality (Hicks et al., 2000; Scekic-Zahirovic et al., 2016), conditional knockout of FUS in motor neurons is not sufficient to cause motor neuron death (Scekic-Zahirovic et al., 2016; Sharma et al., 2016). The effect of depleting FUS on motor neuron morphology was not examined in these studies. Collectively, these findings suggest that FUS misregulation can have both a loss- and gain-of-function effect on neuronal morphology and synapses.

Dysregulation of FUS *in vitro* causes defects in dendritic growth, maturation of spines, and synaptic transmission (Fujii et al., 2005; Fujii and Takumi, 2005; Udagawa et al., 2015; Tibshirani et al., 2017; Qiu et al., 2021). Early work by Fujii et. al. established FUS as an important regulator of dendritic branching, synaptic maturation, and that FUS localizes within dendrites and synapses of primary hippocampal neurons in response to metabatropic glutamate receptor 5 (mGluR5) stimulation (Fujii and Takumi, 2005; Fujii et al., 2005). Moreover, FUSKO neurons have altered dendritic branching and immature dendritic spines (Fujii et al., 2005), down-regulation of Gria1 and AMPA receptor surface expression and reduced miniature EPSC amplitudes (Udagawa et al., 2015). Morphological changes in FUSKO neurons also corresponded with an increased RNA content in the dendrites (Fujii et al., 2005), consistent with the previously discussed functions of FUS in mRNA transport, stability and translation regulation (Fujii and Takumi, 2005; Morlando et al., 2012; Udagawa et al., 2015; Kapeli et al., 2016; Kamelgarn et al., 2018; Sevigny et al., 2020). Importantly, the ability of FUS to traffic within dendrites and synapses was dependant on intact microtubules and actin filaments as determined by the addition of cytochalasin B or nocodazole, inhibitors of the assembly of actin filaments or microtubules, respectively (Fujii et al., 2005).

Expression of ALS/FTD-associated FUS mutants in primary neurons shows that FUS dysregulation has an impact on dendritic branches and spines (Shiihashi et al., 2017; Tibshirani et al., 2017; Qiu et al., 2021). The defects in dendritic growth, branch points, and dendritic length in primary cortical neurons expressing FUSR521C were attributed to splicing defects of *BDNF* mRNA caused by aberrant FUSR521C binding to the 5’ splice junction sequences *Bdnf* mRNA (Qiu et al., 2021). Adding BDNF to these cultures only partially ameliorated defects in dendritic branching (Qiu et al., 2021), suggesting that other factors are contributing to these defects. hFUSΔNLS expression in primary cortical neurons, but not in cultures expressing hFUSWT, show similar defects in dendritic branching, but these changes were attributed to decreased mRNA trafficking and global protein synthesis in dendrites (Shiihashi et al., 2017). Dendritic attrition is also observed in primary motor neurons expressing hFUSR521G, but not in neurons expressing hFUSWT (Tibshirani et al., 2017). In this model, the primary branches closest to the soma of the motor neurons expressing hFUSR521G were spared, however, intermediate and terminal branches were significantly reduced, along with the average number of processes per neuron and total dendrite length (Tibshirani et al., 2017). These changes corresponded with a reduction in nuclear Brg1 expression (Tibshirani et al., 2017), which is an identified interactor of FUS (Chesi et al., 2013). The relevance between FUS dysregulation and expression and function of the nBAF complex remains to be fully elucidated, but the observed changes in nBAF subunits in sporadic ALS and ALS-C9 cases (Tibshirani et al., 2017) suggests important involvement in the disease. Collectively, these studies underscore the impact of transcription and translation and their potential impact on the function and structure of neurons.

### Mechanisms of synaptic dysfunction caused by FUS

Findings from ALS/FTD-FUS patient and disease models show that FUS regulates diverse cellular processes including those involved in synaptic transmission, signaling cascades, cytoskeleton dynamics mitochondrial function and energy metabolism (Schweitzer et al., 2006; Martins-De-Souza et al., 2012; Garone et al., 2020). Unbiased proteomic analysis of frontal cortical tissues from postmortem tissues of FTLD-FUS and controls showed altered expression of proteins associated with synaptic communication (syntaxin subunits: STX1A, STX1B, and STXBP1, synapsin 3: SYN3, synaptotagmin I: SYT1) and those involved in cellular transport pathways (clathrin-associated adaptor proteins: AP1B1, AP2A1, AP2A2, AP2B1) (Martins-De-Souza et al., 2012). These findings were consistent with previously discussed changes in synaptic connectivity observed in FTLD and ALS (Henstridge et al., 2018; Malpetti et al., 2021; Salmon et al., 2021). Additionally, FTLD-FUS tissues had significant changes in proteins involved in signaling pathways (calcium-related proteins, CAMK2A, protein phosphatase 3, catalytic subunit, alpha isozyme: PPP3CA and neurochondrin: NCDN) (Martins-De-Souza et al., 2012). Other proteins with altered expression are implicated in cellular structure and cytoskeleton assembly including subunits of tubulin (TUBB1, TUBB2A, TUBB2C, and TUBA4B), dynamin 2 (DYN2) and neural growth regulator 1 (NEGR1) (Martins-De-Souza et al., 2012). Changed FTLD-FUS proteomic profiles are also associated with energy metabolism and mitochondrial function (glutamate dehydrogenase 2: GLUD2, glucose-6-phosphaste isomerase: GPI, malate dehydrogenase 1 MDH1 and NDUFS1) that are implicated in disease (Schweitzer et al., 2006; Martins-De-Souza et al., 2012). Similar changes were found in FUSP525L and FUSWT human iPSC lines differentiated into ventral spinal motor neurons (Garone et al., 2020). Genome-wide transcriptome analysis of FUS models reflect proteomic changes observed in patients along with a wide-range of altered cellular processes (Nakaya and Maragkakis, 2018; De Santis et al., 2019; Garone et al., 2020; Humphrey et al., 2020; Tsai et al., 2020; Ho et al., 2021). While these changes are likely to impact neuronal function and synaptic connectivity, the mechanistic causes for global changes in protein and gene expression by FUS dysregulation and their relationship to synaptic dysfunction are not known.

ALS/FTD-FUS models provide evidence that misregulation of RNA translation and protein synthesis by FUS underly alterations in neuromorphology and synapses (Sephton et al., 2014; Lopez-Erauskin et al., 2018). Genome-wide expression analysis of spinal cords from pre-symptomatic FUSR521G mice showed no significant changes in pre-mRNA splicing or mRNA expression but had altered protein synthesis at synapses that corresponded with dendritic attrition and fewer mature spines (Sephton et al., 2014). FUSR521C and R521H variants are shown to repress nascent protein production in the processes of primary hippocampal cultures (Lopez-Erauskin et al., 2018) and in axon terminals (Murakami et al., 2015). *In vitro* studies also show that FUS variants alter protein expression levels without significant changes in mRNA expression levels (Nakaya and Maragkakis, 2018; De Santis et al., 2019; Garone et al., 2020; Tsai et al., 2020). Expression of FUS variants in immortalized cell-lines, or in neurons differentiated from mouse embryonic stem cells, caused a decreased expression of mitochondrial encoded proteins without significant changes in overall translation or mRNA expression levels (Nakaya and Maragkakis, 2018; Tsai et al., 2020). FUS variants were found to sequester mRNAs encoding mitochondrial respiratory chain complex (RCC) proteins, causing a reduction in encoded proteins and disruption of mitochondrial networks (Tsai et al., 2020). The impact of these changes on neuronal functions were not assessed. Similarly, proteomic and transcriptomic profiles of isogenic pairs of FUSP525L and FUSWT human iPSC lines differentiated into ventral spinal cord populations showed no correlation between transcript and protein levels, effects that are attributed to selective binding of FUS mutants to the 3’UTRs of target mRNAs (De Santis et al., 2019; Garone et al., 2020). Misregulation of RNA translation and protein synthesis by FUS are shown to occur through different mechanisms including: defects in mRNA trafficking due to aberrant sequestration of RNA in granules or dysfunctional liquid-liquid phase transitions (Birsa et al., 2021), repression of translation through interactions with mRNA or polyribosomes (Udagawa et al., 2015; De Santis et al., 2019; Sevigny et al., 2020), and/or regulation of mRNA stability through non-sense mediated decay (NMD) (Kamelgarn et al., 2018; Ho et al., 2021). Future studies will need to investigate how reported changes in RNA translation and protein synthesis occur in these models and the impact on synaptic function.

FUS is shown to mediate the transport and stabilization of mRNAs important for synaptic function (Fujii and Takumi, 2005; Udagawa et al., 2015; Yokoi et al., 2017). Knocking down FUS in primary neurons leads to fewer mature dendritic spines, reduced miniature EPSC amplitude, and down-regulation of Gria1 expression and AMPA receptor surface expression (Udagawa et al., 2015). FUS was shown to regulate *Gria1* mRNA stability at the 3’UTR and that changes in Gria1 expression were shown to occur through destabilization of *Gria1* mRNA (Udagawa et al., 2015). Reintroduction of Gria1 in FUS knockdown mice partially restored behavioral impairments and in neurons restored synaptic dysfunction (Udagawa et al., 2015), demonstrating that FUS-mediated Gria1 expression is an important component for proper synaptic transmission. FUS and ELAV-like proteins were also shown to cooperatively control *SynGAP* mRNA stability in a 3’UTR length-dependent manner, resulting in a decrease expression of SynGAP α2 (Yokoi et al., 2017). Abnormal spine maturation and FTLD-like behavioral deficits in FUS-knockout mice were partially restored by expression of SynGAPα2 (Yokoi et al., 2017). While these findings provide evidence that FUS destabilization of specific mRNAs can impact synaptic function, it remains unclear whether other mRNA targets of FUS are also contributing to synaptic abnormalities caused by FUS misregulation. To address this question, FusΔNLS/+ mice were used to identify changes in synaptic RNAs (Sahadevan et al., 2021). FusΔNLS/+ mice had an increase in 485 RNAs associated with synaptic function and a decrease in 136 synaptic RNAs associated with cytoskeletal organization and RNA metabolism (Sahadevan et al., 2021). Interestingly, the majority of misregulated synaptic mRNAs in FusΔNLS/+ mice were not identified as synaptic mRNA targets of endogenous mouse FUS (Sahadevan et al., 2021), findings that could reflect the aberrant binding of FUS variants, but not wild-type FUS, to the 3’UTR region of mRNAs (Hoell et al., 2011; Garone et al., 2021). Among those identified as FUS targets, mRNAs with increased stability had FUS binding sites within exonic regions, while those with decreased stability had a higher proportion binding sites within 3’UTRs (Sahadevan et al., 2021). While the alterations in the synaptic RNA profiles in FusΔNLS/+ mice are likely to affect synaptic homeostasis, the direct implicates for these changes require further investigation. Collectively, these studies show that FUS dysfunction leads to alterations in protein translation and expression, however, the detailed mechanisms by which these changes alter neuronal function are not yet known.

## Discussion

The clinical, pathological and genetic overlap between ALS and FTD has provided insights into mechanisms of disease, particularly as they relate to changes in neuronal morphology and synaptic dysfunction. Analysis of patient samples and ALS/FTD models strongly suggest that synaptic dysfunction occurs early in the disease process, before any significant neurodegeneration or cell death. Recent discoveries from proteomic and transcriptomic studies underscore the diverse cellular pathways and biological processes involved in synaptic dysfunction that contribute to the neurodegenerative pathology. Moreover, these studies highlight converging mechanisms of synaptic dysfunction in ALS and FTD. The identification of reliable biomarkers from body fluids or the development of new tools to image synaptic changes in patients may prove useful for early detection and diagnosis. Moreover, concentrated efforts to investigate the pre-symptomatic and early-symptomatic biochemical mechanisms leading to synaptic dysfunction will undoubtedly benefit our understanding of the disease and future therapeutic approaches.

The current body of work examining ALS/FTD-linked C9ORF72, TDP-43, and FUS variants, strongly implicates their involvement in synaptic dysfunction. The emphasis for future studies should be placed on examining the effects of these variants in both pre-symptomatic and symptomatic animal models to better understand the stages of disease progression and their contribution to changes in corticospinal connectivity and loss of synapses. Additionally, more work is needed to better understand the role of non-cell autonomous contributions of other cell types in maintaining dendritic spines and synapses. The current body of work from ALS/FTD-C9 patients and models suggest that C9ORF72 has several important roles in the soma and in distal regions if the neuron. To date, there are no studies that have analyzed the effects of C9ORF72 on axonal or synaptic proteomes and transcriptomes, which may provide additional insights into the mechanisms by which C9ORF72 causes early synaptic dysfunction. TDP-43 and FUS are important regulators of many cellular processes that are implicated in neuromorphology and synaptic homoeostasis through their RNA-binding capabilities. More work is needed to better understand and delineate the primary and secondary mechanisms by which dysregulation of TDP-43 and FUS initiate synaptic loss in both gain-of-function and loss-of-function models.

## Author Contributions

CS: manuscript conceptualization, writing, and figure composition. PD: manuscript writing and editing. PG: literature review of manuscript and Table 1. All authors contributed to the article and approved the submitted version.

## Funding

This work was supported by Brain Canada Future Leader’s Award (to CS), ALS Canada–Brain Canada Hudson Translational Team Grant (to CS), Natural Sciences and Engineering Research Council of Canada Grants RGPIN-2020-06376 and DGECR-2020-00060 (to CS), RGPIN-2018-06227 and DGECR-2018-00093 (to PD), and Fonds de Recherche du Québec Santé (to PD).
